# Comparison of the 10-year outcomes of cemented and cementless unicompartmental knee replacements: data from the National Joint Registry for England, Wales, Northern Ireland and the Isle of Man

**DOI:** 10.1080/17453674.2019.1680924

**Published:** 2019-10-22

**Authors:** Hasan R Mohammad, Gulraj S Matharu, Andrew Judge, David W Murray

**Affiliations:** a Nuffield Department of Orthopaedics, Rheumatology and Musculoskeletal Sciences University of Oxford, Nuffield Orthopaedic Centre, Oxford;;; b Musculoskeletal Research Unit, Bristol Medical School, University of Bristol, Level 1 Learning and Research Building, Southmead Hospital, Bristol, UK

## Abstract

Background and purpose — Unicompartmental knee replacement (UKR) offers advantages over total replacement but has higher revision rates, particularly for aseptic loosening. The cementless Oxford UKR was introduced to address this. We undertook a registry-based matched comparison of cementless and cemented UKRs.

Patients and methods — From 40,552 Oxford UKRs identified by the National Joint Registry for England, Wales, Northern Ireland and Isle of Man (NJR) we propensity score matched, based on patient, surgical, and implant factors, 7,407 cemented and 7,407 cementless UKRs (total = 14,814).

Results — The 10-year cumulative implant survival rates for cementless and cemented UKRs was 93% (95% CI 90–96) and 90% (CI 88–92) respectively, with this difference being significant (HR 0.76; p = 0.002). The risk of revision for aseptic loosening was less than half (p < 0.001) in the cementless (0.42%) compared with the cemented group (1.00%), and the risk of revision also decreased for unexplained pain (to 0.46% from 0.74%; p = 0.03) and lysis (to 0.04% from 0.15%; p = 0.03). However, the risk of revision for periprosthetic fracture increased significantly (p = 0.01) in the cementless (0.26%) compared with the cemented group (0.09%). 10-year patient survival rates were similar (HR 1.2; p = 0.1).

Interpretation — The cementless UKR has improved 10-year implant survival compared with the cemented UKR, independent of patient, implant, and surgical factors. This improved survival in the cementless group was primarily the result of lower revision rate for aseptic loosening, unexplained pain, and lysis, suggesting the fixation of the cementless was superior. However, there was a small increased risk of revision for periprosthetic fracture with the cementless implant.

Over 100,000 primary knee replacements are performed annually in the United Kingdom, with these numbers rapidly increasing (National Joint Registry 2018). This includes both total knee replacement (TKR) and unicompartmental knee replacement (UKR). Although UKR offers significant advantages over TKR including faster recovery, fewer complications, improved function, and lower mortality (Liddle et al. 2014, 2015, Burn et al. 2018), its revision rate is higher in the National Registries (New Zealand Joint Registry 2016, Australian Orthopaedic Association 2018, National Joint Registry 2018).

The most commonly used UKR is the Phase 3 Oxford (Zimmer Biomet, Swindon, UK), which has a fully congruent mobile bearing and is implanted using a minimally invasive approach (Pandit et al. 2006). The cemented version was introduced in 1998. The commonest reasons for revision include aseptic loosening and pain (Mohammad et al. 2018). Radiolucent lines, also known as physiological radiolucencies, are indicative of fibrocartilage at the interface and are seen in over half of cemented UKR tibial components (Gulati et al. 2009). In the presence of pain, these can be misinterpreted as aseptic loosening and lead to revisions despite studies showing no relation (Gulati et al. 2009). In an attempt to decrease the revision rate a cementless version was introduced in 2004, with the only changes to the implant being a porous coating of titanium and hydroxyapatite, and the femoral component having an additional peg.

Randomized controlled trials comparing cemented and cementless UKRs found no statistically significant difference in functional outcomes, but the prevalence of partial and complete radiolucencies was reduced with cementless implants (Pandit et al. 2013). These trials were too small to compare revision rates. However, data from the New Zealand Joint Registry (NZJR) suggest that the cementless UKR has a lower revision rate than the cemented UKR (New Zealand Joint Registry 2016). It is not clear whether the difference in revision rate seen in the NZJR is due to differences in the implants or to other factors. For example it could be that more experienced surgeons, who are doing larger numbers and therefore have lower revision rates, are predominantly using cementless components.

The National Joint Registry for England, Wales, Northern Ireland and Isle of Man (NJR) was established in April 2003 and is now the world’s largest arthroplasty register with over 2 million joint replacements recorded and is linked to the UK’s Office of National Statistics (ONS) mortality data (National Joint Registry 2018). Unfortunately, the NJR does not report the revision rates of cemented and cementless UKR separately in its Annual Report.

We used NJR data to compare the revision rates following cemented and cementless Oxford UKRs. Our null hypothesis was that there would be no difference between cemented and cementless UKR implant survival. To ensure that any difference in implant performance was due to the fixation rather than other factors, we propensity matched cemented and cementless cases on patient, surgeon (including caseload), and implant factors.

## Patients and methods

A retrospective observational study was performed using NJR records and was approved by the NJR Research Sub-Committee (National Joint Registry 2018). The NJR collects data on patient factors (including age, sex, BMI, ASA grade), implant factors (including component design, sizes, and manufacturer type), and surgical factors (surgical approach, cemented or cementless fixation (for femoral and tibial components), indication, caseload, operating surgeon grade) for each replacement procedure, which are provided by the operating surgeon. The NJR database is linked to the Office of National Statistics, which provides data on patient mortality. The NJR achieves high levels of patient consent (93%) and linkability (95%) to subsequent operations (National Joint Registry 2018).

**Figure 1.**
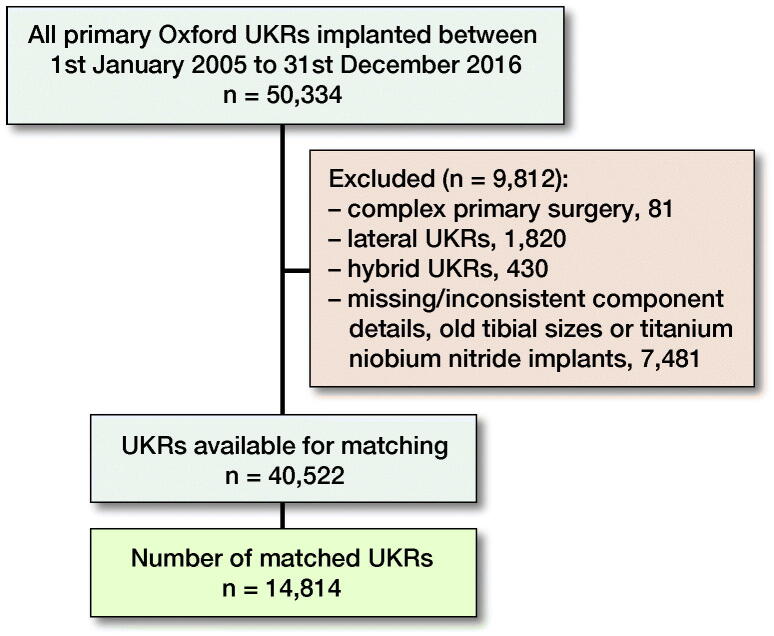
Data flowchart of NJR database cleaning.

Anonymized patient data were extracted from the NJR database which included all primary Oxford UKRs implanted between January 1, 2005 and December 31, 2016 (n = 50,334). After data cleaning involving removal of lateral UKRs, hybrids, complex primaries, old tibial sizes, and missing/inconsistent component missing/inconsistent, there were 40,522 UKRs (30,814 cemented and 9,708 cementless) eligible for study inclusion (Figure 1).

Given the potential for factors other than the type of fixation to affect the revision rate (Prempeh et al. 2008, Memtsoudis et al. 2009, Selby et al. 2012, Judge et al. 2013, Elmallah et al. 2015, Lim et al. 2015, Hamilton et al. 2016, Liddle et al. 2016, Bayliss et al. 2017, Hosaka et al. 2017, Murphy et al. 2018, Picard et al. 2018, Deere et al. 2019, Lenguerrand et al. 2019) we a priori matched the cemented and cementless groups for multiple known confounders using propensity scores. These propensity scores were generated from patient demographics, surgical factors (including surgeon caseload) and implant factors. Surgical factors included surgeon caseload, defined as the average number of UKRs done per year and stratified into low (< 10 cases/year), medium (10 to < 30 cases/year) and high volume (≥ 30 cases/year) as described previously (Liddle et al. 2016). By controlling for these covariates the use of propensity score matching would allow the true effect of implant fixation on revision surgery to be accurately assessed. This a priori approach was supported by the substantial differences in patient, surgical, and implant factors between the unmatched cemented and cementless groups (Table 1, see Supplementary data).

### Statistics

Logistic regression was used to generate a propensity score representing the probability that a patient received a cementless UKR. All patient, surgical, and implant factors in Table 1 (see Supplementary data) were used for matching, apart from BMI, which had a large proportion of missing data. This approach is consistent with previous studies (Matharu et al. 2018a and b), and our data demonstrated BMI was similar between the 2 fixation groups both before and after matching. The algorithm used matched on the logit of the propensity score with a 0.02-SD caliper width. The matching ratio was 1:1. We used greedy matching without replacement. This approach has been shown to have superior performance for estimating treatment effects (Austin 2009). Standardized mean differences (SMDs) were examined both before and after matching to assess for any covariate imbalance between the cemented and cementless UKRs, with SMDs of 10% or more considered suggestive of covariate imbalance (Austin 2009). After matching, 14,814 UKRs (7,407 cemented and 7,407 cementless) were included for analysis.

Outcomes of interest were: (1) implant survival, (2) indications for revision surgery, and (3) patient survival. Cumulative survival was determined using the Kaplan–Meier method. The endpoint for implant survival was revision surgery (any implant component removed, exchanged, or added) and the endpoint for patient survival was mortality. Implant and patient survival rates were compared between the cemented and cementless groups, using Cox regression models, with the proportional hazards assumptions assessed and satisfied in all analyses. A multi-level frailty model was used in the regression models to control for patient clustering within surgeons. Additionally, to account for clustering within the matched cohort, a robust variance estimator was used in regression models. Univariable and adjusted models were also assessed. The adjusted models included covariates with residual imbalance after matching (SMD of 10% or more) (Austin 2009). The proportional chi-square test with Yates’ correction was used to compare the frequency of revisions for specific indications between the cemented and cementless UKR groups.

All statistical analyses were performed using Stata (Version 15.1; StataCorp, College Station, TX, USA) except propensity score matching, which was performed using R (Version 3.4.0; R Foundation for Statistical Computing, Vienna, Austria). P-values of < 0.05 were considered significant, with 95% confidence intervals (CI) presented.

### Ethics, funding, and potential conflicts of interest

This study was based entirely on existing patient records acquired during routine clinical care and thus did not require ethical approval (Wade 2005). This project was fully approved by the NJR Research Sub Committee. This research did not receive any specific grant from funding agencies in the public, commercial, or not for profit sectors. Institutional and Personal funding has been received from Zimmer Biomet.

## Results

The matched cohort included 14,814 UKRs with 7,407 cemented UKRs and 7,407 cementless UKRs. The mean age at surgery was 65 years (SD 9.5), with 6,155 women (42%) and 8,659 men (58%). The mean BMI was 30 (SD 5.0) with the primary indication for surgery being osteoarthritis in 14,633 knees (99%).

Patient, surgical (including caseload), and implant characteristics became well balanced between the cemented and cementless groups after propensity score matching (Table 1, see Supplementary data). The only covariate with residual imbalance was year of primary surgery, which when adjusted for in the regression models did not change the findings presented below.

**Figure 2.**
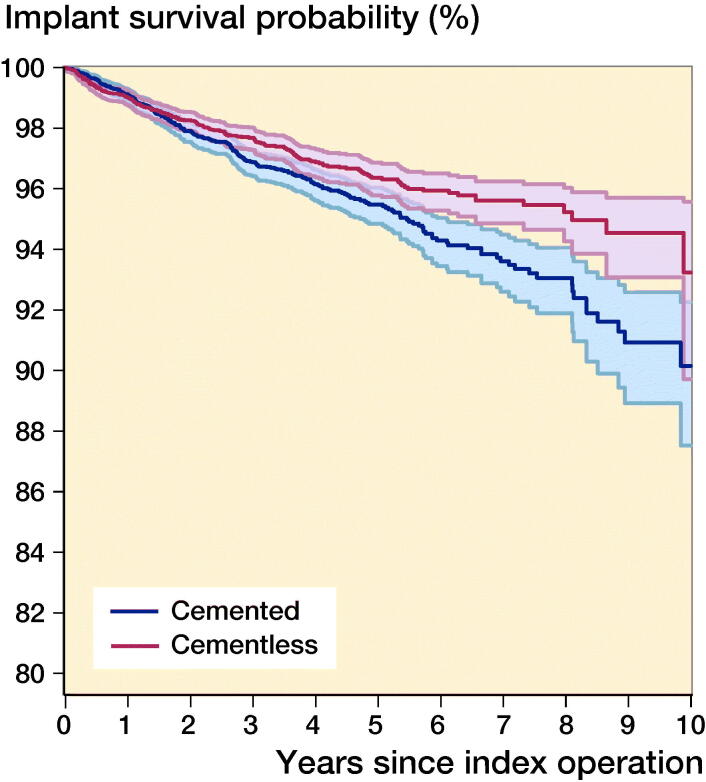
Kaplan–Meier implant survival rates for matched cemented (n = 7,407) and cementless (n = 7,407) UKR implants up to 10 years.

In the matched cohort, the mean follow-up for both cemented and cementless implants was 4 years (SD 2). 507 knees, 218 (2.9%) cementless and 289 (3.9%) cemented, underwent revision surgery. The 10-year cumulative implant survival rates were 93% (CI 90–96) and 90% (CI 88–92) for cementless and cemented respectively (Figure 2). The corresponding cumulative revision rates were 7% (CI 4–10) and 10% (CI 8–13) at 10 years respectively. Cementless UKRs had a significantly reduced revision rate compared with cemented UKRs (HR = 0.76, CI 0.64–0.91; p = 0.002).

**Figure 3.**
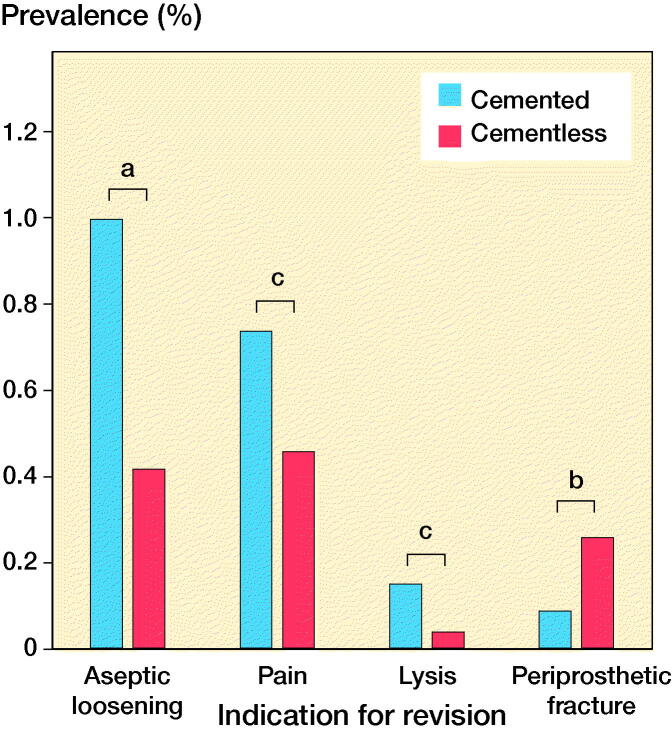
Comparison of the reasons for revision between matched cemented and cementless implants that were statistically significant. **^a^**p < 0.001, **^b^**p = 0.01, and **^c^**p = 0.03 (chi-square test with Yates’ correction).

The most common reasons for revision in the cemented group were aseptic loosening (n = 74, 1.00%), pain (n = 55, 0.74%), and osteoarthritis progression (n = 72, 0.97%) (Table 2, see Supplementary data). In the cementless group the most common reasons for revision were osteoarthritis progression (n = 55, 0.74%), pain (n = 34, 0.46%), and aseptic loosening (n = 31, 0.42%) (Table 2, see Supplementary data). 4 specific revision indications were significantly different between cemented and cementless groups: revision for aseptic loosening was 1.0% versus 0.42% (p < 0.001); for pain 0.74% versus 0.46% (p = 0.03); and for lysis 0.15% versus 0.04% (p = 0.03), respectively (Figure 3). The risk of revision for periprosthetic fracture was significantly higher (p = 0.01) in the cementless group (n = 19, 0.26%) compared with the cemented (n = 7, 0.09%).

**Figure 4.**
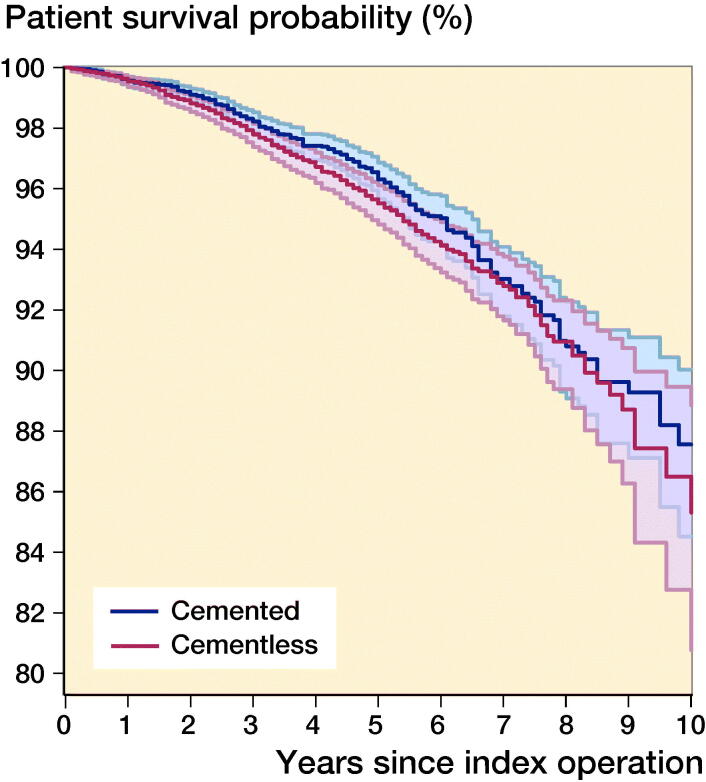
Kaplan–Meier patient survival for matched cemented (n = 7,407) and cementless (n = 7,407) UKR implants up to 10 years.

There were 517 (276 cementless and 241 cemented) patient deaths. 11 deaths occurred within 90 days of surgery (7 cementless and 4 cemented). The cumulative 10-year patient survival rates for cementless UKR were 85% (CI 81–89) and 88% (CI 85–90) for cemented UKR (Figure 4). This difference was not statistically significant (HR = 1.2, CI 0.98–1.4; p = 0.1).

## Discussion

The early cementless UKR had poor outcomes, but the results have improved with time (Bernasek et al. 1988, Lindstrand et al. 1988, Harilainen et al. 1993, Bergenudd 1995, Mohammad et al. 2018). Data from the NZJR has shown that the revision rate of the cementless Oxford is substantially less than the cemented. However, as this is raw unmatched data, we cannot be certain why this is, and many factors such as more experienced surgeons tending to use the cementless implant may contribute. We have now shown in approximately 15,000 UKRs that are propensity matched to exclude other potential patient, surgical (including surgeon caseload), and implant confounders that the revision rate of the cementless Oxford UKR is 24% less (HR 0.76, p = 0.002) than the cemented out to 10 years. This therefore suggests that the cementless Oxford UKR is a better performing implant than the cemented.

The primary reason for this difference is that the rates of revision for aseptic loosening, pain, and lysis were all substantially lower in the cementless group. Indeed, the combined revision rate from these causes was about half for cementless compared with cemented fixation. Previous randomized studies have shown the incidence of narrow tibial radiolucent lines (otherwise known as physiological radiolucencies) is much lower with cementless rather than cemented fixation, suggesting that the fixation is much better (Kendrick et al. 2015). This would explain why the revision rate for aseptic loosening and lysis has decreased. It is, however, not clear why the revision rate for pain has decreased. It could be that the incidence of pain is less with cementless fixation. Alternatively it could be that in the presence of pain surgeons are more likely to revise a component that has a radiolucent line, even though the evidence would suggest that the radiolucent line is not a cause of pain and is not indicative of loosening (Gulati et al. 2009).

The only reason for revision that occurred statistically significantly more frequently with the cementless than the cemented group was peri-prosthetic fracture, with the rates of revision being respectively 0.26% and 0.09% (p = 0.01). The difference is 0.17% which is relatively small compared with the decrease in revision rate. Furthermore, the mean time to revision for peri-prosthetic fracture was one year, which is much earlier than for most other revisions, so with time and increased follow-up the proportion of revisions that are due to peri-prosthetic fracture should decrease further. Information concerning the site of the peri-prosthetic fracture is not recorded, but it is likely that the majority were tibial plateau fractures. A cadaver study has shown that the load to fracture is lower with a cementless rather than a cemented tibial component (Seeger et al. 2012), suggesting that the increased rate of fracture may relate to the interference fit between the cementless tibial component and the impaction required to implant it. As tibial plateau fractures are major complications, often requiring revision TKR with stems and wedges, surgeons should take care to avoid them when implanting cementless components. In particular they should avoid deep cuts, make the vertical cut just medial to the tibial spine, protect the posterior cortex, ensure the tibial trial can be inserted with finger pressure, and impact the tibial component with care and a light hammer.

We believe that this is the first large-scale study of any type of knee replacement which has demonstrated that the cementless version has lower revision rates than the cemented, and that the difference is due to improved fixation. This may, however, relate to the design of the implant. As all ligaments are preserved and there is an unconstrained mobile bearing the loads transmitted to the bone–implant interface are predominantly compressive with minimal shear or tension, which is ideal for cementless fixation. The results may therefore not apply to all types of UKR or to TKR.

The main study limitation is that it is based on Registry data and is therefore a study of revision and not reoperation or other clinical outcomes. However, many of these other outcomes have already been studied in randomized trials. Additionally, the reasons for revision in the NJR are those recorded at the time of surgery even if this subsequently changed due to histopathology and microbiology data. Registries can underreport revisions (Sabah et al. 2015, 2016) although there is no reason to believe this would differ between the groups, and it is not possible to confirm causality in registry-based studies. Another limitation is that, despite matching, there is potential for residual confounding and matching can reduce the generalizability of our findings. The groups were not perfectly matched given there was imbalance in the year of primary surgery, as the cementless components were introduced after the cemented. This was mitigated as many of the early cemented cases were excluded as they had different shaped tibial components (numerical sizes). Although operating techniques improve with time, there were no differences in our findings when we adjusted the regression models for year of primary surgery. There was a substantial proportion of BMI data missing so we did not match on BMI. However, the BMI distribution between cemented and cementless UKR was the same both before and after propensity matching. The only way to achieve balance with respect to both known and unknown confounders is with a randomized trial. However, to compare revision rates would require very large numbers and long follow-up, which would be impractical; we believe propensity matching offers the next best alternative.

In conclusion, in this propensity matched registry-based study, the observed risk of revision of the cementless Oxford UKR was 24% less than that of the cemented out to 10 years. This was primarily because the risk of revision for aseptic loosening, pain, and lysis all decreased in the cementless, suggesting that it is due to improved fixation.

### Supplementary data

Tables 1 and 2 are available as supplementary data in the online version of this article, http://dx.doi.org/10.1080/17453674. 2019.1680924

## Supplementary Material

Supplemental Material
