# Supplementary material for: Comparison of the 10-year outcomes of cemented and cementless unicompartmental knee replacements: data from the National Joint Registry for England, Wales, Northern Ireland and the Isle of Man
Source: Acta Orthop. 2019 Oct 22;91(1):76–81. doi: 10.1080/17453674.2019.1680924 (PMC7006803; doi:10.1080/17453674.2019.1680924)
Supplement: Supplemental Material [file IORT_A_1680924_SM3038.pdf]

Supplementary data

Table 1. Patient and surgical factors before and after propensity score matching. Values are frequency (%) unless otherwise specified

Covariate	Unmatched cohort All UKRs n = 40,522	Unmatched cohort Cemented 76% n = 30,814	Unmatched cohort Cementless 24% n = 9,708	SMD	Matched cohort All UKRs n = 14,814	Matched cohort Cemented 50% n = 7,407	Matched cohort Cementless 50% n = 7,407	SMD
Sex								
Female	18,775 (46.3)	14,707 (47.7)	4,068 (41.9)	0.12	6,155 (41.6)	3,077 (41.5)	3,078 (41.6)	< 0.001
Male	21,747 (53.7)	16,107 (52.3)	5,640 (58.1)		8,659 (58.4)	4,330 (58.5)	4,329 (58.4)	
Age at surgery								
Mean (SD)	64.7 (9.5)	64.7 (9.5)	64.8 (9.5)	0.01	64.7 (9.5)	64.6 (9.6)	64.7 (9.5)	0.003
BMI (cases)	26,966	18,669	8,297		11,801	5,565	6,236	
Mean (SD)	30.2 (5.0)	30.2 (5.0)	30.4 (5.2)	0.04	30.3 (5.0)	30.2 (4.9)	30.4 (5.2)	0.05
Primary diagnosis								
Primary OA	40,059 (98.9)	30,474 (98.9)	9,585 (98.7)	0.02	14,633 (98.8)	7,314 (98.7)	7,319 (98.8)	0.006
Other	463 (1.1)	340 (1.1)	123 (1.3)		181 (1.2)	93 (1.3)	88 (1.2)	
Bilateral UKRs	1,325 (3.3)	874 (2.8)	451 (4.6)	0.1	493 (3.3)	245 (3.3)	248 (3.4)	0.002
ASA grade								
1	8,441 (20.8)	6,321 (20.5)	2,120 (21.8)	0.05	3,025 (20.4)	1,536 (20.7)	1,489 (20.1)	0.02
2	28,687 (70.8)	21,983 (71.3)	6,704 (69.1)		10,499 (70.9)	5,227 (70.6)	5,272 (71.2)	
3 or above	3,394 (8.4)	2,510 (8.1)	884 (9.1)		1,290 (8.7)	644 (8.7)	646 (8.7)	
VTE—chemical								
LMWH (± other)	23,789 (58.7)	17,561 (57.0)	6,228 (64.2)	0.40	9,311 (62.9)	4,624 (62.4)	4,687 (63.3)	0.02
Aspirin only	5,158 (12.7)	4,152 (13.5)	1,006 (10.4)		1,446 (9.8)	727 (9.8)	719 (9.7)	
Other	7,747 (19.1)	5,496 (17.8)	2,251 (23.2)		3,641 (24.6)	1,851 (25.0)	1,790 (24.2)	
None	3,828 (9.5)	3,605 (11.7)	223 (2.3)		416 (2.8)	205 (2.8)	211 (2.8)	
VTE—mechanical								
Any	38,947 (96.1)	29,316 (95.1)	9,631 (99.2)	0.25	14,662 (99.0)	7,332 (99.0)	7,330 (99.0)	0.003
None	1,575 (3.9)	1,498 (4.9)	77 (0.8)		152 (1.0)	75 (1.0)	77 (1.0)	
Year of surgery								
2005	1,108 (2.7)	1,100 (3.6)	8 (0.1)	1.32	17 (0.1)	9 (0.1)	8 (0.1)	0.18
2006	1,929 (4.8)	1,889 (6.1)	40 (0.4)		78 (0.5)	38 (0.5)	40 (0.5)	
2007	2,730 (6.7)	2,702 (8.8)	28 (0.3)		89 (0.6)	61 (0.8)	28 (0.4)	
2008	3,426 (8.5)	3,344 (10.9)	82 (0.8)		229 (1.6)	147 (2.0)	82 (1.1)	
2009	3,721 (9.2)	3,460 (11.2)	261 (2.7)		499 (3.4)	238 (3.2)	261 (3.5)	
2010	3,660 (9.0)	3,256 (10.6)	404 (4.2)		752 (5.1)	349 (4.7)	403 (5.4)	
2011	3,652 (9.0)	3,013 (9.8)	639 (6.6)		1,054 (7.1)	417 (5.6)	637 (8.6)	
2012	3,680 (9.1)	2,962 (9.6)	718 (7.4)		1,400 (9.5)	695 (9.4)	705 (9.5)	
2013	3,582 (8.8)	2,622 (8.5)	960 (9.9)		1,860 (12.6)	996 (13.4)	864 (11.7)	
2014	4,182 (10.3)	2,637 (8.6)	1,545 (15.9)		2,762 (18.6)	1,500 (20.3)	1,262 (17.0)	
2015	4,303 (10.6)	2,112 (6.9)	2,191 (22.6)		3,083 (20.8)	1,528 (20.6)	1,555 (21.0)	
2016	4,549 (11.2)	1,717 (5.6)	2,832 (29.2)		2,991 (20.2)	1,429 (19.3)	1,562 (21.1)	
Surgeon grade								
Consultant	36,346 (89.7)	27,775 (90.1)	8,571 (88.3)	0.06	13,310 (89.8)	6,688 (90.3)	6,622 (89.4)	0.03
Other	4,176 (10.3)	3,039 (9.9)	1,137 (11.7)		1,504 (10.2)	719 (9.7)	785 (10.6)	
Surgeon caseload/year								
< 10	16,130 (39.8)	13,474 (43.7)	2,656 (27.4)	0.43	4,691 (31.7)	2,327 (31.4)	2,364 (31.9)	0.01
10 to < 30	16,785 (41.4)	12,685 (41.2)	4,100 (42.2)		6,664 (45.0)	3,336 (45.0)	3,328 (44.9)	
≥ 30	7,607 (18.8)	4,655 (15.1)	2,952 (30.4)		3,459 (23.4)	1,744 (23.5)	1,715 (23.2)	
Surgical approach								
Medial parapatellar	37,052 (91.4)	28,154 (91.4)	8,898 (91.7)	0.01	13,649 (92.1)	6,827 (92.2)	6,822 (92.1)	0.003
Other	3,470 (8.6)	2,660 (8.6)	810 (8.3)		1,165 (7.9)	580 (7.8)	585 (7.9)	
Minimally invasive surgery								
No	21,076 (52.0)	16,287 (52.9)	4,789 (49.3)	0.07	7,600 (51.3)	3,796 (51.3)	3,804 (51.4)	0.002
Yes	19,446 (48.0)	14,527 (47.1)	4,919 (50.7)		7,214 (48.7)	3,611 (48.8)	3,603 (48.6)	
Femoral component size								
Extra small	89 (0.2)	47 (0.2)	42 (0.4)	0.14	47 (0.3)	26 (0.4)	21 (0.3)	0.02
Small	9,408 (23.2)	6,904 (22.4)	2,504 (25.8)		3,479 (23.5)	1,752 (23.7)	1,727 (23.3)	
Medium	21,214 (52.4)	16,608 (53.9)	4,606 (47.4)		7,280 (49.1)	3,617 (48.8)	3,663 (49.5)	
Large	9,700 (23.9)	7,171 (23.3)	2,529 (26.1)		3,970 (26.8)	1,990 (26.9)	1,980 (26.7)	
Extra large	111 (0.3)	84 (0.3)	27 (0.3)		38 (0.3)	22 (0.3)	16 (0.2)	

Table 1. Continued.

Covariate	All UKRs n = 40,522	Unmatched cohort Cemented 76% n = 30,814	Cementless 24% n = 9,708	SMD	All UKRs n = 14,814	Matched cohort Cemented 50% n = 7,407	Cementless 50% n = 7,407	SMD
Tibial component size								
AA	130 (0.3)	93 (0.3)	37 (0.4)	0.37	58 (0.4)	29 (0.4)	29 (0.4)	0.01
A	3,805 (9.4)	3,453 (11.2)	352 (3.6)		679 (4.6)	336 (4.5)	343 (4.6)	
B	9,158 (22.6)	7,288 (23.7)	1,870 (19.3)		2,994 (20.2)	1,513 (20.4)	1,481 (20.0)	
C	11,576 (28.6)	8,769 (28.5)	2,807 (28.9)		4,284 (28.9)	2,137 (28.9)	2,147 (29.0)	
D	9,668 (23.9)	7,098 (23.0)	2,570 (26.5)		3,965 (26.8)	1,974 (26.7)	1,991 (26.9)	
E	4,753 (11.7)	3,216 (10.4)	1,537 (15.8)		2,179 (14.7)	1,095 (14.8)	1,084 (14.6)	
F	1,432 (3.5)	897 (2.9)	535 (5.5)		655 (4.4)	323 (4.4)	332 (4.5)	
Bearing type								
Anatomic	32,708 (80.7)	23,301 (75.6)	9,407 (96.9)	0.65	14,198(95.8)	7,092 (95.8)	7,106 (95.9)	0.009
Symmetric	7,814 (19.3)	7,513 (24.4)	301 (3.1)		616 (4.2)	315 (4.3)	301 (4.1)	
Bearing size								
3	9,269 (22.9)	6,226 (20.3)	3,003 (30.9)	0.37	4,056 (27.4)	2,056 (27.8)	2,000 (27.0)	0.02
4	16,219 (40.0)	12,126 (39.4)	4,093 (42.2)		6,288 (42.5)	3,128 (42.2)	3,160 (42.7)	
5	8,552 (21.1)	6,765 (22.0)	1,787 (18.4)		2,942 (19.9)	1,459 (19.7)	1,483 (20.0)	
6	3,846 (9.5)	3,268 (10.6)	578 (6.0)		1,042 (7.0)	519 (7.0)	523 (7.1)	
7	1,667 (4.1)	1,506 (4.9)	161 (1.7)		306 (2.1)	150 (2.0)	156 (2.1)	
8	620 (1.5)	563 (1.8)	57 (0.6)		118 (0.8)	62 (0.8)	56 (0.8)	
9	349 (0.9)	320 (1.0)	29 (0.3)		62 (0.4)	33 (0.4)	29 (0.4)	
Bone graft								
No	40,374 (99.6)	30,745 (99.8)	9,629 (99.2)	0.08	14,758 (99.6)	7,377 (99.6)	7,381 (99.7)	0.009
Yes	148 (0.4)	69 (0.2)	79 (0.8)		56 (0.4)	30 (0.4)	26 (0.4)	

Abbreviations: ASA = American Society of Anesthesiologists score, BMI = body mass index, OA = osteoarthritis, SD = standard deviation, SMD = standardized mean difference, UKR = unicompartmental knee replacement, VTE = venous thromboembolism.

Table 2. Reasons for revision in the matched cohort

Revision indication	All UKRs (n = 14,814) n (%)	Cemented UKRs (n = 7,407) n (%)	Time to revision indication mean (SD)	Cementless UKRs (n = 7,407) n (%)	Time to revision indication mean (SD)	p-value [a]
Aseptic loosening [b]	105 (0.71)	74 (1.00)	1.5 (2.1)	31 (0.42)	2.0 (2.0)	< 0.001
OA progression	127 (0.86)	72 (0.97)	3.5 (2.5)	55 (0.74)	3.7 (2.0)	0.1
Pain [b]	89 (0.60)	55 (0.74)	2.8 (2.1)	34 (0.46)	2.0 (1.5)	0.03
Other	70 (0.47)	41 (0.55)	2.5 (2.0)	29 (0.39)	2.0 (1.3)	0.2
Dislocation/subluxation	49 (0.33)	28 (0.38)	1.6 (1.6)	21 (0.28)	1.2 (1.2)	0.4
Instability	33 (0.22)	13 (0.18)	2.9 (1.3)	20 (0.27)	1.9 (1.9)	0.3
Component dissociation	29 (0.20)	13 (0.18)	1.4 (1.1)	16 (0.22)	2.2 (2.1)	0.6
Malalignment	37 (0.25)	13 (0.18)	1.2 (0.9)	24 (0.32)	1.8 (1.4)	0.1
Infection	28 (0.19)	15 (0.20)	1.9 (2.1)	13 (0.18)	1.8 (1.8)	0.8
Periprosthetic fracture [b]	26 (0.18)	7 (0.09)	1.0 (0.9)	19 (0.26)	1.0 (2.4)	0.01
Lysis [b]	14 (0.09)	11 (0.15)	2.5 (1.4)	3 (0.04)	1.6 (1.0)	0.03
Wear	14 (0.09)	7 (0.09)	4.0 (2.8)	7 (0.09)	3.5 (2.9)	1.0
Stiffness	12 (0.08)	5 (0.07)	1.6 (0.6)	7 (0.09)	1.4 (1.0)	0.7
Implant fracture	1 (0)	1 (0.01)	2.0	0 (0)	N/A	No revisions
Patellar wear	0 (0)	0 (0)	N/A	0 (0)	N/A	No revisions
Tibial wear	0 (0)	0 (0)	N/A	0 (0)	N/A	No revisions
Incorrect sizing	0 (0)	0 (0)	N/A	0 (0)	N/A	No revisions
Patellar mal tracking	0 (0)	0 (0)	N/A	0 (0)	N/A	No revisions

[a] Comparisons between the frequency of revision indications were conducted using the chi-square test.
[b] Refers to revision indications that were statistically significantly different between cementless and cemented implants.
Abbreviations: OA = osteoarthritis, UKR = unicompartmental knee replacement.
